# Effectiveness of Testing and Contact-Tracing to Counter COVID-19 Pandemic: Designed Experiments of Agent-Based Simulation

**DOI:** 10.3390/healthcare9060625

**Published:** 2021-05-24

**Authors:** Young Jin Kim, Pyung-Hoi Koo

**Affiliations:** Department of Systems Management and Engineering, Pukyong National University, Busan 48513, Korea; youngk@pknu.ac.kr

**Keywords:** COVID-19, infectious disease outbreak, testing and contact tracing, agent-based model, designed experiments

## Abstract

The widespread outbreak of the novel coronavirus disease COVID-19 has posed an enormous threat to global public health. A different set of policy interventions has been implemented to mitigate the spread in most countries. While the use of personal protective equipment and social distancing has been specifically emphasized, South Korea has deployed massive testing and contact-tracing program from the early stage of the outbreak. This study aims at investigating the effectiveness of testing and contact-tracing to counter the spread of infectious diseases. Based on the SEICR (susceptible-exposed-infectious-confirmed-recovered) model, an agent-based simulation model is developed to represent the behavior of disease spreading with the consideration of testing and contact-tracing in place. Designed experiments are conducted to verify the effects of testing and contact tracing on the peak number of infections. It has been observed that testing combined with contact tracing may lower the peak infections to a great extent, and it can thus be avoided for the hospital bed capacity to be overwhelmed by infected patients. It is implied that an adequate capability of testing and contact-tracing may enable us to become better prepared for an impending risk of infectious diseases.

## 1. Introduction

Since the first reported outbreak in December 2019, the novel coronavirus disease COVID-19 has swept all over the world and posed a significant threat to global public health. As of April 2021, more than 130 million confirmed cases have been reported, which accounts for about 1.7% of the world population [1]. COVID-19 has a covert characteristic in that some infected individuals show no or minor symptoms, but they may still spread the disease. Policy interventions to mitigate the transmission of highly infectious diseases like COVID-19 may include three different types of measures: protection, isolation, and immunization. The use of personal protective equipment (PPE) and social distancing may be referred to as the protection measure. In contrast, testing and contact-tracing can be the isolation measure to identify and detain those who had close contact with the infected individuals as well as the infected themselves. An immunization can be pursued with the deployment of effective vaccines. While working at an extraordinary pace to develop therapeutics and vaccines against COVID-19, non-pharmaceutical interventions of protection and isolation have been implemented to varying degrees in different countries until pharmaceutical interventions become available.

South Korea, witnessing the first local outbreak of COVID-19 in late January 2020, has adopted the intervention measures of massive testing and contact tracing to control the epidemic country-wide from the early stage of the outbreak. The idea behind testing and contact tracing is that the infected individuals are tested as early as possible and then isolated from others to prevent further disease transmission. Recognizing that the early detection of infections through tests is imperative to counter the highly infectious disease, South Korea has implemented innovative, large-scale testing facilities, including drive-through screening stations and temporary screening office and worked closely with the private sector to secure an adequate supply of tests from the onset of the pandemic [2,3]. Following the confirmed positive test, the contact-tracing process is initiated to identify and test people who may have been exposed to the disease to prevent onward transmission. Personal interviews with the infected individuals, the GPS information from their cell phones, transaction records from credit and debit cards, and personal identification QR codes at high-traffic locations are used for contact tracing [3]. The contact-traced individuals are then messaged and asked to be tested and stay at home in self-quarantine for two weeks to separate themselves from others to minimize the possibility of exposing other people to infection should they are infected. Besides, whenever a new confirmed case is identified in a district, websites and smartphone apps distribute hour-by-hour timelines of the infected. People who think they may have been in the same place with the infectious are urged to report to testing centers [4]. With testing and contact-tracing programs despite the potential risk of privacy infringement, South Korea managed the epidemic relatively well without such extreme measures adopted by many other countries as closing borders, closing businesses, and restricting travels. As of 30 March 2021, 0.2% of the population is reported to be infected in South Korea, which is remarkably lower than the OECD countries’ average of 5.5% [1].

Traditionally, mathematical models are widely used to examine the transmission behavior of an infectious disease. Most of the mathematical models are compartmental ones, with the population divided into classes and assumptions being made about the rate of transition from one class to another [5]. The susceptible-infectious-recovered (SIR) model is one of the simplest compartmental models for the spread of infectious disease, where the population is divided into susceptible, infectious, and recovered classes [6]. There are various modifications to the SIR model. They include the susceptible-infectious-susceptible (SIS) model where there is no immunity upon recovery, the susceptible-infectious-recovered-deceased (SIRD) model, which considers the deceased compartment as well, susceptible-infectious-quarantine-susceptible (SIQS) model with quarantine compartment, and the susceptible-exposed-infectious-recovered (SEIR) where a latent period of the disease is considered [7,8]. Even though well accepted and widely applied, these traditional models may not account for the transmission dynamics of COVID-19 pandemic where non-pharmaceutical interventions including social distancing, testing, contact tracing, quarantine, and isolation are widely in place to mitigate the spread of disease. References [9,10] proposed the susceptible-exposed-infectious-confirmed-recovered (SEICR) model for the COVID-19 spread where testing and case isolation are included. Reference [8] introduced the SIR-X model that includes public containment and quarantine. It is argued that contact tracing and the delay between symptom onset and case isolation are critical factors to reduce the spread of disease [11,12]. Studies in [13] show the effectiveness of stratified testing where people in groups that are more likely to be exposed to the virus get tested regularly. One distinct approach about contact tracing is proposed in [14], where bidirectional contact tracing is proposed. The reverse-tracing is also used to identify the parent case who infected a known case and then continue tracing to iteratively discover other cases related to the parent case. Reference [15] presents a mathematical modeling study to investigate the effectiveness of testing, contact tracing, isolation, and social distancing. They argue that combining isolation and contact tracing would reduce transmission more than mass testing or self-isolation alone. Although the mathematical models give a general idea about the behavior of disease spreading, they have some limitations to consider details on the heterogeneity of the population and the interactions of people at an individual level, which are essential characteristics for the virus spread in the world under epidemic.

With the capability to mimic complex systems involving uncertainty and explicitly addressing interactions among individuals in the system, the agent-based modeling (ABM) approach may seem a promising alternative. ABM is a computational model representing complex systems composed of autonomous individual agents or entities that can interact with each other under the same system environment [16,17]. It has drawn increasing attention to simulating the spread of infectious diseases such as HIV [18], avian influenza [19,20], malaria [21], and influenza [22,23]. Recently, the ABM approach has also been employed to investigate the spread of COVID-19. Reference [24] analyzed the effects of non-pharmaceutical interventions, such as lockdown, isolation, and the use of face masks, to contain the spread of COVID-19, and economic impacts of the disease are also investigated. Reference [25] developed an ABM model to investigate the impacts of control strategies including educational center closing, social distancing, and office closure, on controlling the COVID-19 outbreak in Urmia, Iran. Reference [26] presented an ABM simulation model where infection probabilities and mobility restrictions are included. Reference [27] examined the impact of testing, contact tracing, and quarantine on COVID-19 transmission in Boston metropolitan area under different intervention scenarios. Reference [28] proposed an ABM platform to simulate the spreading of COVID-19 in which the effect of school closure, lock-down, and different levels of social distancing can be examined. In addition, their model is used to explore different testing approaches, such as hospital testing and drive-through testing, and vaccination strategies.

When an individual has a symptom and gets tested, it takes a certain amount of time (defined as test turn-in time) to have the test results back. The individuals who take the screening test may move around until they get the test results, which may transmit the virus to others. When an individual is confirmed positive, two lines of intervention activities take place: case isolation and contact tracing. The COVID-19 positive individuals are hospitalized or isolated from others so that they do not spread the virus any further. Along with the case isolation, contact chains of the test-positive individuals are traced, and those who have been in contact with the infected individual are tested and self-quarantined for a certain amount of time. If an individual identified by the contact tracing process is tested positive, a new contact-tracing process is again initiated. The importance of contact tracing is highlighted by its impact on the transmission by asymptomatic patients. Different from the symptomatic patients who show various symptoms, such as fever and coughing, the asymptomatic patients do not exhibit symptoms at any time during the course of infection. Studies suggest that people infected with COVID-19 can transmit the virus whether they have symptoms or not. Asymptomatic individuals are not likely to get tested because they have no symptoms, so that they infect others while they are infectious. Transmission through asymptomatic patients could only be prevented by tracing contacts of the confirmed case and testing those contacts. For better effectiveness of contact tracing, the reduction of delay between symptom onset and case confirmation (case isolation) is critical because the infectious patients can infect others without knowing their own infection during this time. The isolation delay is composed of two factors, testing delay and test turn-in time. To the best of the authors’ knowledge, no existing studies explore the combined effect of testing and test turn-in time affecting isolation, contact tracing, and quarantine, which can play a pivotal role in mitigating the spread of COVID-19. This study presents an ABM simulation model to emulate the dynamic behavior of COVID-19. Non-pharmaceutical interventions such as testing, test result confirmation, contact tracing, isolation, and quarantine are explicitly included in the model at an individual level. The NetLogo 6.2.0 platform [29] is used to design and implement the ABM model. It is widely acknowledged that the NetLogo program provides a simple yet powerful modeling capacity, mobility of individuals with local interactions in a grid space, built-in graphical interfaces, and rich documentation [30]. Based on the ABM model developed, the conventional three-level factorial design has been employed to examine interaction effects of testing and contact tracing as well as their own main effects. A popular response surface methodology (RSM) has been applied to investigate the impact of testing and contact-tracing on the peak number of infections, which is critical to the proper management of hospital bed capacity to accommodate infected patients from the early stage of epidemics. The RSM is known as a suitable analysis tool, particularly in situations where several input variables potentially influence some performance measures [31].

## 2. Methods of Model Development

**Figure 1:**
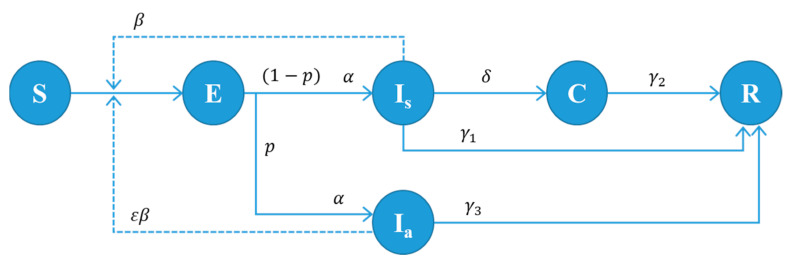
State transition diagram of our model. Every individual should be in one of the states, S, E, I_s_, I_a_, C, or R. The parameters in the figure are defined as follows: β: disease transmission rate of symptomatic patients. ε: relative infectiousness of asymptomatic patients. α: progression rate from state E to I_a_ or I_s_ (1/α: incubation period); p: percentage of the asymptomatic case; δ: test turn-in rate (1/δ: time between testing and result confirmation). $\gamma_{1}$: recovery rate for infected but not tested individuals ($1/\gamma_{1}$: infectious period for non-tested infected patients). $\gamma_{2}$: recovery rate for symptomatic and test-confirmed individuals ($1/\gamma_{2}$: isolation period for test-confirmed patients). $\gamma_{3}$: recovery rate for asymptomatic individuals ($1/\gamma_{3}$: infectious period for asymptomatic patients).

The proposed model is based on the SEICR model with some modifications to include the unique characteristics of COVID-19. The population is divided into six different groups: susceptible (S), exposed (E), infectious symptomatic before test confirmation (I_s_), infectious asymptomatic (I_a_), infectious isolated with test positive (C), and removed (recovered or dead) (R). The state transition diagram is given in Figure 1 with the transition probability between states. The population size N is the sum of class sizes: N = S + E + I_s_ + I_a_ + C + R. The state of individuals changes over time according to the transition probabilities. No births and deaths are assumed because the time horizon is relatively short compared to the human lifespan, apart from the death caused by COVID-19. A virus-infected person should be in one of the states, E, I_s_, I_a_, and C. However, only individuals under states I_s_ or I_a_ are infectious.

The dynamics of infection in the SEICR-based model can be expressed by a set of ordinary differential equations (ODEs) (see [6] for ODEs for various infectious cases). The ODEs can give answers to simple questions about the behavior of disease spreading. However, it often fails to consider essential aspects of the population’s heterogeneous behavior and social interaction. This paper introduces an ABM simulation model based on the state transition diagram given in Figure 1, where testing and contact tracing activities are explicitly included.

The ABM simulation model is initially set up with a certain number of people in the susceptible state S and the remainder in the exposed state E. Individuals in state E may become infectious after some period (so-called incubation period) and move to an infectious state according to the progression rate. The virus may be transmitted to susceptible individuals from infectious ones with the transmission rate whenever they contact each other. The susceptible who are exposed to the virus then move from state S to state E. The infectious individuals are divided into two classes based on the onset of the symptoms: symptomatic (I_s_) and asymptomatic (I_a_). Studies suggest that asymptomatic individuals are also infectious, but less likely to transmit the virus than symptomatic individuals [32,33,34].

**Figure 2:**
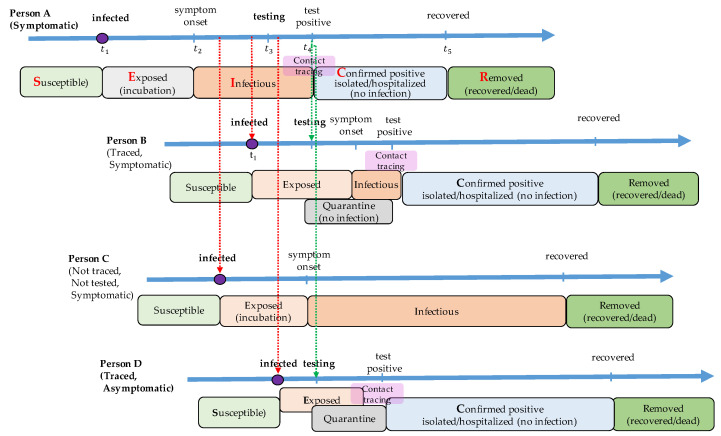
An example of testing and contact tracing process with a symptomatic individual. A susceptible individual (Person A) is exposed to the virus at time $t_{1}$. After an incubation time, he or she shows symptoms at $t_{2}$. A screening test is carried out at $t_{3}$, and a test result is confirmed at $t_{4}$. When the test result is positive, he or she is isolated from others, and the contact tracing process is initiated. Persons B and D are traced from the contact tracing process, and they are tested and quarantined for 14 days no matter what the test results are. Even though Person B and Person D get infected, they may not transmit the disease anymore once the contact is traced. On the other hand, person C, who has not been traced, does not know that he or she is infected and possibly transmits the virus to others. When Person B (or Person D) is confirmed positive through the screening test, a new contact tracing process is initiated.

**Figure 3:**
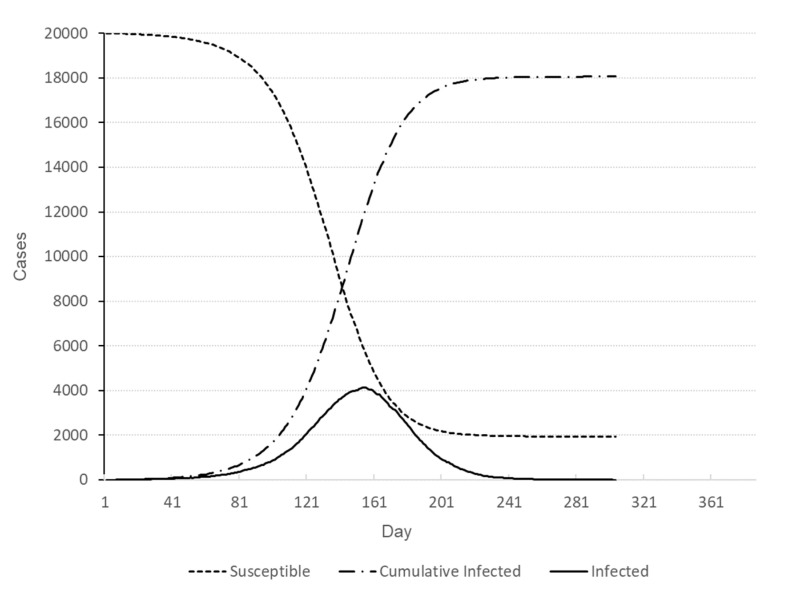
Number of individuals in different states for baseline scenario.

COVID-19 diagnostic testing identifies current infection at an individual level and is performed when a person has symptoms of infection. Asymptomatic individuals may not get tested because they have no symptoms. Even some of the symptomatic individuals do not recognize their infection without the screening test until their recovery. The non-tested infectious individuals remain in state I_s_ or I_a_, and travel freely to spread the disease to the susceptible. The proposed model assumes that all the asymptomatic patients may not get tested while some of the symptomatic individuals get tested to check if they are infected. The testing coverage is defined as the proportion of symptomatic individuals who get tested before recovery. Figure 2 shows the disease transmission process from a symptomatic patient. Person A, who is infected at time $t_{1}$, has no symptoms during the incubation period until time $t_{2}$. After the onset of symptoms, Person A gets a diagnostic test. It takes a certain amount of time (so-called turn-in time) to have the test results back. The infectious state of Person A starts at $t_{2}$ and ends at $t_{4}$. When an individual is tested positive, two kinds of intervention activities take place: case isolation and contact tracing. Person A will be in state C where he or she is hospitalized or isolated from others at time $t_{4}$ so that he or she does not spread the virus any further. The contact tracing process is initiated to locate those who have been in contact with the newly confirmed case. Then, the contacts get tested and self-quarantined for a certain amount of time. It is seen that the infected individuals identified by the contact tracing process (Persons B and D in Figure 3) shortly remain in state I, which may lead to less disease transmission. New contact tracing processes are then again initiated with cases found by the contact tracing process with Person A. The individuals in state I without test (Person C in Figure 2) and those in state C with test (Persons A, B, and D in Figure 3) recover from the disease and move to state R (recovered or dead) after a certain amount of time, and they remain immune to the disease from then on.

It is critical to set up the agent-based simulation model with appropriate epidemiological parameters and associated variables to better describe the behavior of disease spreading. The proposed model’s baseline values are obtained from various sources such as previous studies, public reports, and our own estimations. The baseline values for the epidemiological parameters are summarized in Table 1. It is assumed that 20% of all infected individuals exhibit no symptoms during their infectious period, as in [12]. Asymptomatic cases are further assumed to be 50% less infectious than symptomatic ones as in [14,27,34].

Critical parameters of infectious disease spread include the incubation and infectious period. While the incubation period refers to the time period from the exposure to the virus to the onset of symptoms, the infectious period is the time period during which the infected individuals can spread the virus to others. Previous studies indicate that the incubation period of COVID-19 is well fitted to a log-normal distribution [14,35,36] with the average ranging from 4 to 6 days [25,37], while the infectious period of symptomatic individuals may follow gamma distribution [38,39,40] with the average of 8 days [8,24,25]. Based on these studies, the proposed model assumes that the incubation period follows a log-normal distribution with mean and standard deviation of 5.5 days and 2.1 days, respectively, and the infectious period follows a gamma distribution with mean and standard deviation of 8.0 days and 2.0 days, respectively.

The *basic* reproduction number, denoted by $R_{0}$, refers to the expected number of secondary infections directly generated by a single case in a population, where all individuals are susceptible to infection and no policy interventions have been adopted. Hence, $R_{0}$ can be thought of as the intrinsic characteristic of an epidemic virus. When $R_{0}>1.0$, the number of infected individuals among the population increases over time. The $R_{0}$ is different from the *effective* reproduction number, denoted $R_{t}$, which is the number of cases generated in the environment at time *t* where some policy interventions including social distancing and face-mask wearing are applied. Several reports indicate that estimates of the basic reproduction number of COVID-19 range from 1.9 to 6.5 [31,37,40,41]. Our model assumes the basic reproduction number of 2.5 as in [14,27] which lead to *β* = 2.48% in our model where *β* is the probability of disease transmission from a symptomatic individual to a susceptible one when they are in contact with each other.

The turn-in time of test results is defined as the amount of time to get the results of the COVID-19 test. Reducing the turn-in time is essential because case isolation and contact tracing may be initiated with the confirmed test results. A few studies distribute information about the turn-in time, reporting that it typically takes from one to three days to get test results back, and it may take longer due to possible backlogs in the lab [42,43]. The effects of turn-in time on disease spreading are also investigated for different values of turn-in time ranging from one to three days. Finally, based on the current COVID-19 outbreak statistics, the fatality rate of COVID-19 is assumed to be 2.2% [1].

The model described above is implemented in the NetLogo 6.2.0 platform. It is assumed that the population size is set to 20,000 among which 20 individuals are being exposed to COVID-19. Initially, the individuals are randomly placed in the space divided into 2809 square grids (53 × 53 patches), and they move around in a random way. In our model, contact is defined as the presence of individuals in the same square grid at the same time slot. The contact is implemented in NetLogo by using a link agent connecting infectious individuals and susceptible ones in the same grid. Later, the links may be used for the contact tracing process. Using a population-based survey result of epidemiologically relevant social contacts, it is indicated that the average number of contacts per day per person is 14–16, with significantly high dispersion [44,45]. The number of daily contacts in our model follows a Poisson distribution with mean 14.2 individuals and a standard deviation 3.8 individuals. In our ABM model, the values for relevant epidemiological parameters can easily be adjusted, so that simulation experiments are performed under various scenarios. The flexibility and interactivity of the ABM in the NetLogo platform enable us to examine the impacts of parameters and control measures on disease spreading under diverse circumstances.

## 3. Designed Experiments of the ABM Simulation and Results

This study employs the conventional 3-level factorial design to examine the effectiveness of testing and contact tracing to counter the spread of COVID-19. Three factors are considered: testing coverage, turn-in time of test results, and contact-tracing ratio, denoted by Factors A, B, and C, respectively. The testing coverage indicates that only a certain percentage of infected individuals may take the test, which clearly affects the spread. It is reported that 40–60% of infected people have mild or no symptoms and may pass the virus to others without being tested [46,47]. The simulation experiment employed the factor setting of 20%, 40%, and 60% for testing coverage, meaning that only the corresponding percentage of infected individuals take the test. Considering that most asymptomatic patients and some of the symptomatic patients may not take the COVID-19 test, the factor setting of the testing coverage may be regarded as a conservative estimate. The spread may also be influenced by the turn-in time of test results, which is dependent upon testing capacity. The factor setting of 1, 2, and 3 days is used in the simulation experiment. Finally, it is argued that, despite the risk of privacy infringement, contact tracing can be quite effective in identifying potentially infectious individuals who had been in close proximity to the infectious. Contact tracing can be complicated due to laws and regulations for privacy protection. It is hardly plausible to conduct a complete and accurate tracing of contact chains, and no dependable estimates are available up to this point. According to the COVID-19 contact tracing dashboard maintained by the Department of Health of the State of New Jersey, USA, nearly 30% of contacts followed up have been provided [48]. The factor setting for contact tracing is set to 20%, 40%, and 60% for the simulation experiment, and thus the corresponding percentage of contacts of confirmed cases will be traced. Table 2 summarizes the factor settings of the simulation experiment.

Simulation runs are performed on a desktop computer with an Intel^®^ Core™ i7-9700 CPU @ 3.00GHz. It takes 12.5 s on average to a single simulation run. The full factorial design with three factors with three levels each yields 27 treatment combinations, and five replications have been made for each treatment combination resulting in 135 simulation runs. Five responses have been recorded for each run; duration of the outbreak (*Y*_1_), number of deaths (*Y*_2_), cumulative number of infections (*Y*_3_), peak number of infections (*Y*_4_), and time of peak infections (*Y*_5_). The experimental design and average responses at each design point are presented in Table 3. For the sake of comparison, the baseline scenario with no testing nor contact-tracing has been run ten times, whose results for five responses are presented in Table 4. A representative baseline profile of the outbreak is depicted in Figure 3.

## 4. Discussion on Statistical Analysis

**Figure 4:**
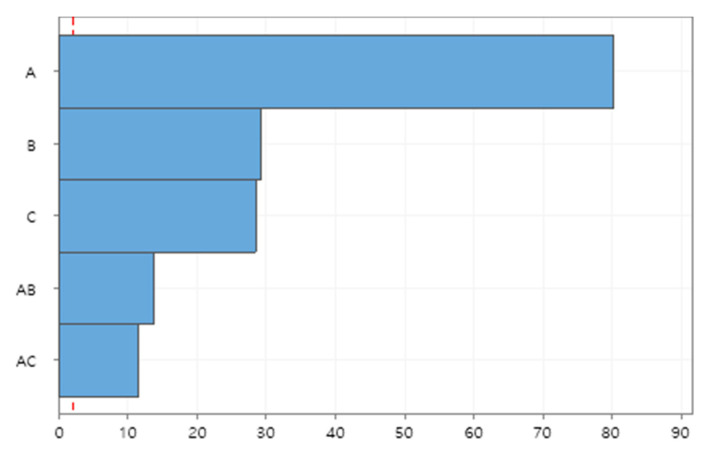
Pareto chart of standardized effects on peak number of infections.

The simulated data have been statistically analyzed with response surface methodology (RSM) using MINITAB Release 19. Investigated are the main effects of individual factors and their interaction effects on the peak number of infections, which is one of the most important aspects for the proper management of hospital bed capacity to deal with infected patients. Figure 4 depicts the Pareto chart of standardized effects of individual factors and their interactions on the peak number of infections. The testing coverage (Factor A) has the most significant impact on the peak number of infections, which is followed by the turn-in time of test results (Factor B) and contact tracing ratio (Factor C). The testing coverage also significantly interacts with the turn-in time (AB) and contact tracing ratio (AC) to affect the peak number, implying that testing and contact tracing complement each other to effectively reduce the transmission of diseases.

**Figure 5:**
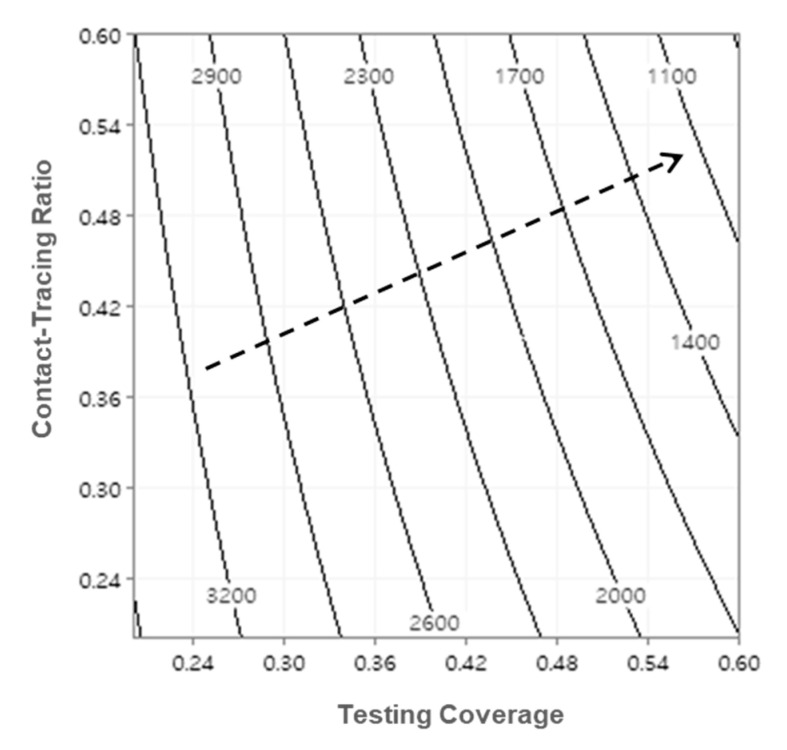
Contour plot of peak number of infections (turn-in time = 1-day).

The functional relationship between factors and responses may also be estimated in a polynomial equation with RSM. The peak number of infections can be estimated by the polynomial equation, of which coefficients are summarized in Table 5, and the adjusted coefficient of determination is 98.4%. Figure 5 depicts the contour plot of the peak number of infections with respect to testing coverage and contact-tracing ratio, while the turn-in time of test results is set to one day. The dashed arrow indicates the direction of steepest descent for the peak number of infections which implies that expanding testing capability alone may not be as effective without widespread contact tracing and vice versa.

**Figure 6:**
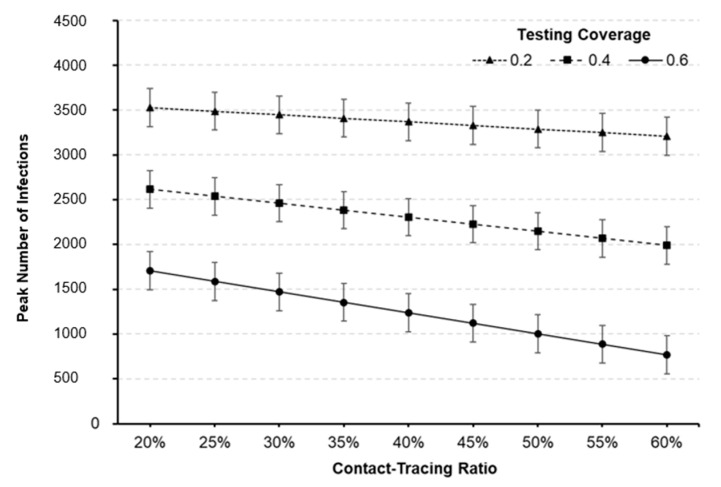
Predicted peak number of infections against testing coverage and contact-tracing ratio (turn-in time = 1-day). The 95% prediction intervals are represented as bar graphs at each point.

**Figure 7:**
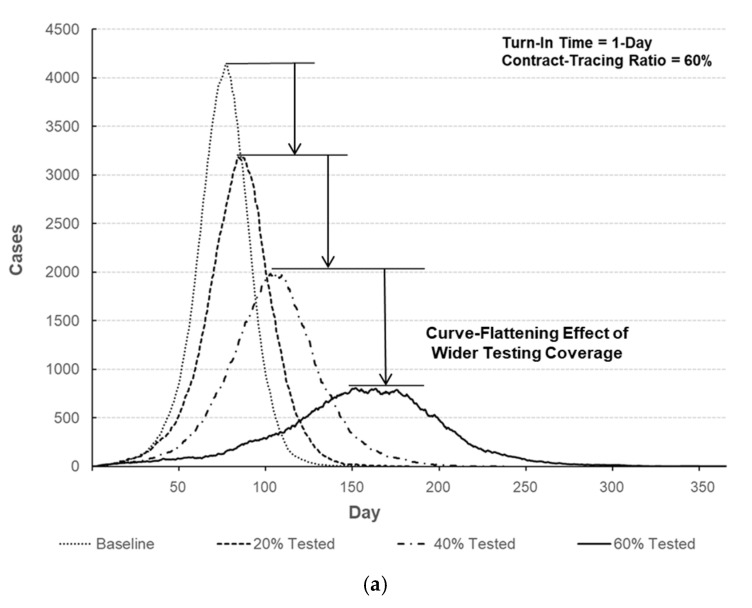

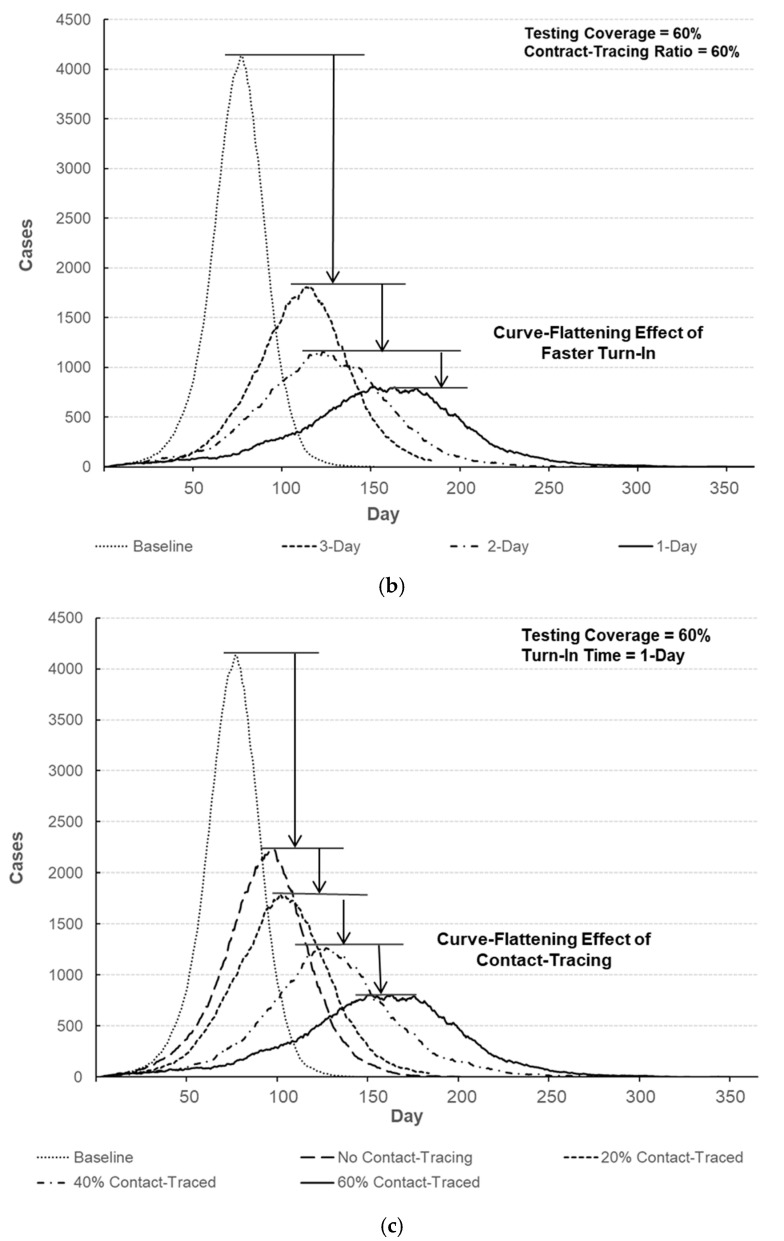
Comparison of the number of infected individuals over time. (**a**) Number of infected individuals for different testing coverages (turn-in time = 1-day, contact-tracing ratio = 60%). (**b**) Number of infected individuals with respect to turn-in time (testing coverage = 60%, contact-tracing ratio = 60%). (**c**) Number of infected individuals for different contact-tracing ratios (turn-in time = 1-day, testing coverage = 60%).

Useful predictions can also be made within the region of experimentation. The peak number of infections with respect to testing coverage and contact-tracing ratio is predicted and depicted in Figure 6. For example, the predicted peak number of infections for factor setting of (A, B, C) = (60%, 1-Day, 60%) is 770.2 with the standard error of 28.3, which yields the 95% prediction interval of (557.1, 983.3). Compared to the baseline scenario given in Table 4, the peak number may be remarkably reduced by 81.4% (= (4145.1−770.2) /4145.1). Outbreak profiles for various scenarios can also be generated and compared to investigate the effectiveness of testing and contact-tracing for curve-flattening. Figure 7 compares the number of infected individuals for different factor settings to that of baseline scenario. It should be noted that the peak number of infections can dramatically be lowered, and thus a shortage of hospital beds and staff may be avoided by implementing testing and contact-tracing programs.

**Figure 8:**
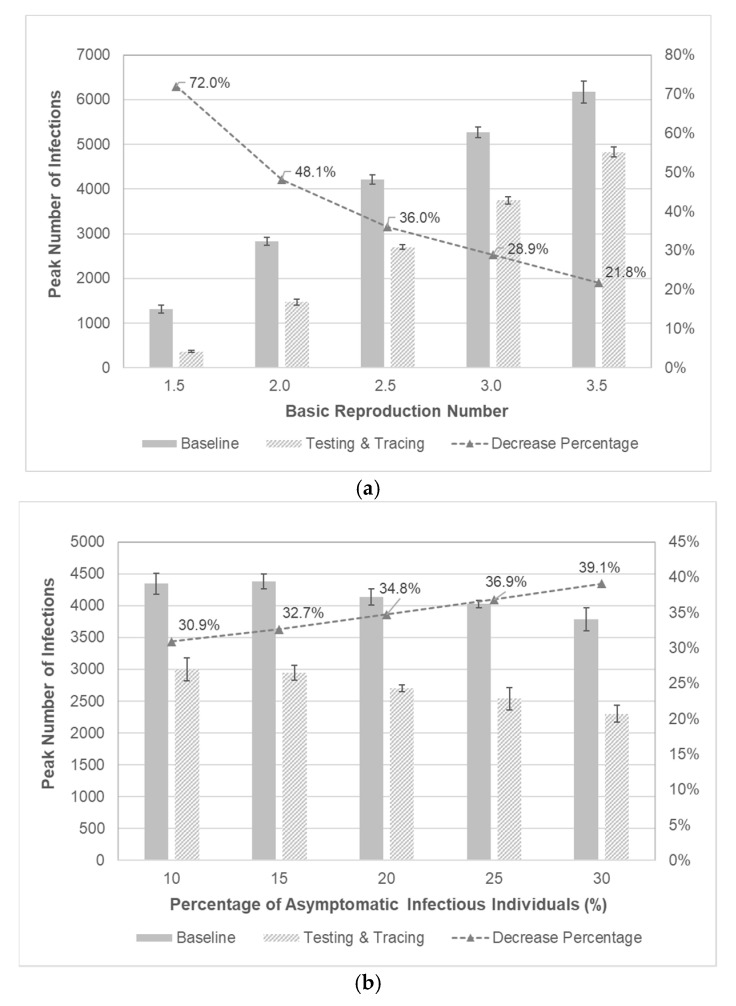
Sensitivity analysis with respect to epidemiological parameters. (**a**) Comparison of peak number of infections with respect to $R_{0}$. The 95% confidence intervals are represented as bar graphs at each point. (**b**) Comparison of peak number of infections with respect to the percentage of asymptomatic infectious individuals. The 95% confidence intervals are represented as bar graphs at each point.

As noted earlier, the spreading behaviors of infectious diseases are highly dependent upon epidemiological parameters. It is elusive and probably quite rare, however, to attain enough information on these parameters, and only *a posteriori* estimates of them may be partially obtained if available. Sensitivity analysis can be conducted to better understand the disease spreading with respect to these parameters. It is intuitive that the basic reproduction number $R_{0}$ significantly affects the disease spreading. The effectiveness of testing and contact tracing is examined for different values of $R_{0}$. The peak infections of baseline scenario are compared to those of factor setting of (A, B, C) = (40%, 2-day, 40%) for different values of basic reproduction number. It is shown in Figure 8a that the implementation of testing and contact-tracing may significantly lower the peak number of infections regardless of basic reproduction number. Observing the decrease percentage of peak infections, testing and contact-tracing may seem specifically effective for smaller values of basic reproduction number. Finally, asymptomatic infectious individuals may easily spread the disease without knowing their infections. The effects of testing and contact-tracing are examined for different values of percentage of asymptomatic cases as shown in Figure 8b. Compared to the baseline scenario, the peak number of infections with testing and contact-tracing is much lower and the decrease percentage tends to be higher when there are more asymptomatic infectious individuals. It is implied that testing combined with contact-tracing can effectively locate and then isolate asymptomatic cases to prevent further spread by innocent ignorance.

## 5. Conclusions

A wide variety of policy interventions to cope with a serious and imminent threat from COVID-19 has been adopted by most countries. While working at an unprecedented pace to develop pharmaceutical measures of vaccines and therapeutics to stop the pandemic, protective measures such as the use of PPE and social distancing have been strongly emphasized to slow the disease spread in the meantime. South Korea has supplemented the policy interventions with massive testing and contact tracing from the early stage of the local outbreak. Whereas aforementioned protective measures have been investigated by a few previous studies, the effectiveness of testing and contract tracing has hardly been dealt with. An agent-based modeling approach has been proposed to examine the effects of testing and contact-tracing program on the disease spread. The simulation model is constructed on the basis of the SEICR model. A set of designed experiments is conducted with three main factors, testing coverage, turn-in time of test results, and contact-tracing ratio. Since closely related to the management of hospital bed capacity, the peak number of infections for various factor settings is analyzed using response surface methodology. As expected, testing and contact tracing may be highly effective for curve-flattening. It is also worth noting that interaction between testing and contact-tracing ratio exhibits a significant effect, implying that expanding testing capability alone may not be as effective without extensive contact-tracing and vice versa.

The main contribution of this study may be described in two folds. First, the proposed simulation model may provide useful insight into the spreading behavior of infectious diseases under various circumstances. Furthermore, examining the impacts of different mitigation measures can be helpful when planning and implementing public health policies to cope with potential outbreaks. To the best of the authors’ knowledge, for example, the effects of turn-in time of test results have not been investigated in previous studies. It is suggested that faster turn-in can be as effective as contact tracing to slow the spread. Therefore, additional policy efforts may be placed on enhancing the community’s testing capability instead of focusing on potentially controversial contact tracing. Second, designed experiments are quite popular to investigate causal relationships in many application areas. This study proposes employing the principles of experimental design combined with agent-based modeling in epidemiological studies. The outbreak of an infectious disease is a highly complicated phenomenon, and it can be challenging to sufficiently explain the dynamics of disease spreading. With the increase in computing power, an agent-based modeling approach has drawn increasing attention for modeling complex systems, and it can be efficiently employed to better describe the behavior of disease spreading. It is expected that more research efforts should be placed on developing flexible and versatile epidemic models to better represent the reality of policy interventions

## Figures and Tables

**Table 1:** Parameters of the ABM simulation and their baseline values.

Parameter	Value	Remark	Reference
% asymptomatic patients	20%		[12]
Relative infectiousness of asymptomatic patients	50%		[14,27,34]
Incubation period	Log-normal distribution with mean 5.5 and SD ^1^ 2.1	Reciprocal of the progression rate	[14,25,35,36,37]
Infectious period	Gamma distribution with mean 8 and SD 2.0	-	[8,24,25,38,39,40]
Basic reproduction number ($R_{0}$)	2.5	-	[14,27]
Transmission rate/contact	2.48%	Estimated from $R_{0}$	
Turn-in time of test results	1~3 days		[28,29]
Fatality rate	2.2%		[1]

^1^ SD: standard deviation.

**Table 2:** Factor settings of simulation experiment.

Levels	Testing Coverage (%) (Factor A)	Turn-in Time (Day) (Factor B)	Contact-Tracing Ratio (%) (Factor C)
low	20	1	20
medium	40	2	40
high	60	3	60

**Table 3:** Simulation results for each treatment combination.

Factor A	Factor B	Factor C	$Y_{1}$	$Y_{2}$	$Y_{3}$	$Y_{4}$	$Y_{5}$
20%	1	20%	171.6	391.0	17,293.4	3516.6	80.8
20%	1	40%	175.4	385.6	17,162.8	3322.8	82.8
20%	1	60%	180.8	368.0	16,828.2	3160.2	83.2
20%	2	20%	176.0	379.6	17,445.2	3581.0	81.0
20%	2	40%	166.2	370.8	17,380.8	3497.8	82.0
20%	2	60%	171.2	382.4	17,129.6	3315.6	81.0
20%	3	20%	169.8	374.0	17,618.8	3784.6	76.6
20%	3	40%	168.6	393.2	17,446.2	3593.8	77.6
20%	3	60%	178.6	394.0	17,315.0	3433.0	80.0
40%	1	20%	192.4	360.2	15,946.8	2694.4	86.2
40%	1	40%	216.0	339.0	15,380.4	2401.4	96.4
40%	1	60%	243.2	331.2	14,575.8	1984.4	102.0
40%	2	20%	173.6	364.2	16,452.6	2993.2	81.4
40%	2	40%	200.0	332.6	15,962.8	2698.2	88.4
40%	2	60%	214.2	335.6	15,523.8	2471.6	93.2
40%	3	20%	177.4	376.0	17,026.0	3338.2	80.0
40%	3	40%	184.6	363.4	16,529.2	2921.2	85.6
40%	3	60%	209.6	348.2	16,164.6	2739.0	89.0
60%	1	20%	245.4	300.4	13,549.6	1673.4	106.4
60%	1	40%	278.6	251.6	11,818.6	1196.8	123.4
60%	1	60%	347.6	216.8	9816.0	694.0	155.0
60%	2	20%	217.4	335.6	14,964.2	2175.6	100.8
60%	2	40%	238.8	304.0	13,687.2	1764.6	105.0
60%	2	60%	278.6	264.8	12,159.6	1261.0	117.0
60%	3	20%	196.6	342.0	15,953.6	2654.6	90.6
60%	3	40%	213.8	330.2	14,991.2	2215.2	97.4
60%	3	60%	240.6	296.0	13,786.2	1722.8	105.8

**Table 4:** Summary of simulation results for baseline scenario.

Summary	$Y_{1}$	$Y_{2}$	$Y_{3}$	$Y_{4}$	$Y_{5}$
Average	152.9	397.4	18,122.5	4145.1	74.0
Standard Error	3.3	3.5	11.4	27.2	0.6
95% Confidence Interval	(145.4, 160.4)	(389.6, 405.2)	(18,096.7, 18,148.3)	(4083.5, 4206.7)	(72.7, 75.3)

**Table 5:** Coefficients of polynomial equation for peak number of infections.

Term	Coefficient	Standard Error of Coefficient	T-Value
Constant	2622.4	8.94	293.17 ***
A	−880.4	11.0	−80.36 ***
B	319.9	11.0	29.20 ***
C	−312.8	11.0	−28.55 ***
AB	184.7	13.4	13.77 ***
AC	−154.4	13.4	−11.50 ***

*** *p*-Value < 0.001.

## Data Availability

Not applicable.
